# Establishing the Top 10 Research Priorities for Adolescent and Young Adult (AYA) Cancer in Canada: A Protocol for a James Lind Alliance Priority Setting Partnership

**DOI:** 10.3390/curroncol31050219

**Published:** 2024-05-17

**Authors:** Perri R. Tutelman, Chantale Thurston, Tamara Rader, Brianna Henry, Tristyn Ranger, Mohamed Abdelaal, Michelle Blue, Timothy W. Buckland, Stefanie Del Gobbo, Lexy Dobson, Emily Gallant, Cheryl Heykoop, Mackenzie Jansen, Lorna Larsen, Nicole Maseja, Sapna Oberoi, Vinesha Ramasamy, Marlie Smith, Evan Taylor, Nadia Wendowsky, Sara Beattie, Jacqueline Bender, Kathryn A. Birnie, Sheila N. Garland, Lindsay Jibb, Melanie Noel, Fiona S. M. Schulte

**Affiliations:** 1 Department of Oncology, University of Calgary, Calgary, AB T2N 1N4, Canada; perri.tutelman@ucalgary.ca (P.R.T.); brianna.henry@ucalgary.ca (B.H.); tristyn.ranger@ucalgary.ca (T.R.); sara.beattie@albertahealthservices.ca (S.B.); 2 Tom Baker Cancer Centre, Calgary, AB T2N 4N2, Canada; 3 Patient Partner, Canadacheryl.1heykoop@royalroads.ca (C.H.); teamshan@gmail.com (L.L.); nmaseja@ucalgary.ca (N.M.);; 4 James Lind Alliance, Southampton SO16 7NS, UK; tamarar@cadth.ca; 5 Department of Medicine, University of Ottawa, Ottawa, ON K1H 8M5, Canada; mabdelaal@toh.ca; 6 Cross Cancer Institute, Edmonton, AB T6G 1Z2, Canada; michelle.blue@albertahealthservices.ca; 7 BC Cancer Centre, Vancouver, BC V5Z 4E6, Canada; 8 CancerCare Manitoba, Winnipeg, MB R3E 0V9, Canada; mjansen@cancercare.mb.ca (M.J.); soberoi@cancercare.mb.ca (S.O.); 9 Princess Margaret Cancer Centre, Toronto, ON M5G 2C4, Canadajackie.bender@uhn.ca (J.B.); 10 School of Social Work and Human Services, University of the Fraser Valley, Abbotsford, BC V2S 7M8, Canada; evan.taylor@ufv.ca; 11 Lawrence Bloomberg Faculty of Nursing, University of Toronto, Toronto, ON M5R 0A3, Canada; lindsay.jibb@sickkids.ca; 12 Department of Anesthesiology, Perioperative and Pain Medicine, University of Calgary, Calgary, AB T2N 1N4, Canada; kathryn.birnie@ucalgary.ca; 13 Department of Psychology, Memorial University, St. John’s, NL A1C 5S7, Canada; sheila.garland@mun.ca; 14 Department of Psychology, University of Calgary, Calgary, AB T2N 1N4, Canada; melanie.noel@ucalgary.ca

**Keywords:** adolescent and young adult, cancer, AYA, priority-setting partnership, research priorities, James Lind Alliance, patient involvement

## Abstract

Adolescents and young adults (AYAs; 15–39 years) diagnosed with cancer have unique medical and psychosocial needs. These needs could be better addressed through research that is focused on the topics that matter most to them. However, there is currently no patient-oriented research agenda for AYA cancer in Canada. This manuscript describes the early development and project protocol for a priority-setting partnership (PSP) for establishing the top 10 research priorities for AYA cancer in Canada. This project follows the PSP methodology outlined by the James Lind Alliance (JLA) to engage patients, caregivers, and clinicians in research prioritization. The steps of a JLA PSP include establishing a steering group and project partners, gathering uncertainties, data processing and verifying uncertainties, interim priority setting, and a final priority setting workshop. The AYA cancer PSP will result in a top 10 list of research priorities identified by Canadian AYA patients, caregivers, and clinicians that will be published and shared broadly with the research community. The first steering group meeting was held in April 2023, and the project is ongoing. The establishment of a patient-oriented research agenda for AYA cancer will catalyze a long-term and impactful research focus and ultimately improve outcomes for AYA patients with cancer in Canada.

## 1. Introduction

Each year, over 9000 Canadian adolescents and young adults (AYAs; ages 15–39 years [1]) are diagnosed with cancer [2]. AYAs are increasingly being recognized as an oncology population with distinct medical (e.g., unique tumour biology, diagnostic delays, poor access to clinical trials, disparities in survival rates) and psychosocial (e.g., social, emotional, and financial impacts) needs [3,4,5]. While initiatives have aimed to improve outcomes and experiences for AYAs in Canada over the last several decades [6,7], their cancer care needs remain largely unmet and unaddressed [8,9,10], likely due to a combination of informational, system, and sociobehavioural barriers [3,11,12]. Rigorous, clinically relevant research in areas that matter most to patients, caregivers, and clinicians is needed to improve outcomes for Canadian AYAs with cancer. 

Research prioritization activities have emerged as a mechanism for engaging knowledge users (i.e., individuals who are likely to use scientific research to make informed decisions about health practices, policies, and/or programs [13]) in establishing patient-oriented research agendas that address the research questions most relevant to a community. Mounting evidence suggests that research that is co-created by patients is more relevant, of higher quality, and associated with better incorporation into clinical practice [14,15]. The James Lind Alliance (JLA) is a non-profit organization that established a systematic process of identifying and prioritizing the key questions that specific knowledge users (e.g., patients, caregivers, and clinicians) want answered by research [16]. JLA priority-setting partnerships (PSPs) ultimately result in a final list of the “top 10” research questions that are primed for action by researchers, funding agencies, patient organizations, and policy makers [16]. There is currently no patient-oriented research agenda specific to AYA cancer in Canada.

The overarching objective of this project is to establish a patient-, caregiver-, and clinician-informed research agenda for AYA cancer in Canada using the James Lind Alliance research prioritization methodology. The specific objectives are to (1) gather data on the uncertainties (i.e., possible research priorities) of AYA patients, caregivers, and clinicians that have not already been answered by research; (2) order questions into a top 10 list of research priorities; and (3) mobilize the research priorities for addressal by scientists, policy makers, and funders. This manuscript describes the early development and project protocol for the AYA Cancer PSP.

## 2. Materials and Methods

**Figure 1:**
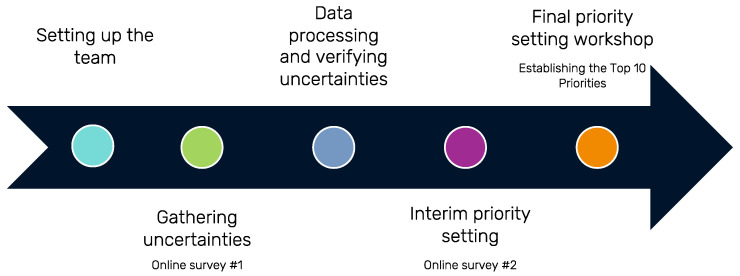
Summary of the James Lind Alliance Priority-Setting Partnership process.

This project follows the PSP methodology established by the JLA (Figure 1). The JLA PSP method follows the guiding principles of equal involvement of clinicians and people with lived experience; inclusiveness; transparency; and a commitment to using and contributing to the research evidence base [16]. This project was approved by the Health Research Ethics Board of Alberta—Cancer Committee (HREBA.CC-23-0156). 

### 2.1. PSP Governance and Team

#### 2.1.1. Project Co-Leads

**Figure 2:**
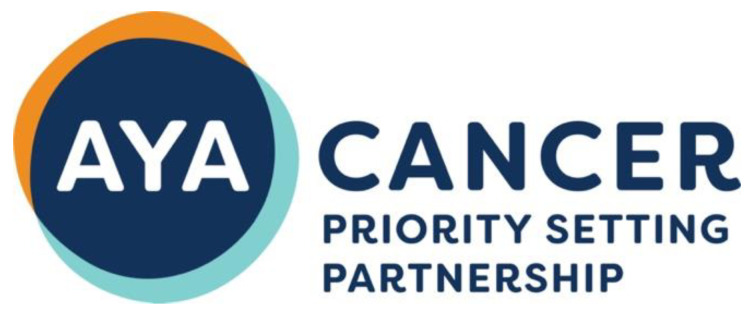
The AYA Cancer Priority-Setting Partnership project logo.

The initial idea for a PSP for AYA cancer in Canada was generated by the lead author, P.T., who approached C.T., patient advocate and Board Chair of AYA CAN (a peer-led national organization advocating for AYAs in Canada affected by cancer), to co-lead the project, together with F.S. The early steps of the PSP prior to the establishment of the steering group (e.g., project branding; Figure 2) were developed collaboratively by the co-leads. 

#### 2.1.2. The PSP Steering Group

JLA PSPs are governed by an expert steering group comprising up to 16 patients, caregivers, and clinicians with relevant expertise on the topic of the PSP [16]. The AYA Cancer PSP steering group is chaired by a JLA adviser (T.R.), supported by a project coordinator (B.H.), and meets monthly to oversee all activities of the PSP, including approving the study objectives, guiding the methods and outcomes for each step, and disseminating the results. The steering group was convened by the project co-leads (P.T., C.T., and F.S.). A call for expressions of interest in joining the steering group was launched in January 2023 and disseminated through social media and the PSP’s network of community organizations (see section below). The call was put out in both English and French and open for approximately 2 weeks. A total of 78 applications were received. Of the applications received, 56% were from individuals who identified as AYA patients or caregivers, 23% were from individuals who identified as clinicians, 9% were from individuals who identified as individuals with multiple roles, and 12% were from individuals who did not meet the eligibility criteria (i.e., researchers and patients outside the AYA age range at diagnosis). Selection of members for the steering group was based on ensuring a roughly equal balance of patient/caregiver and clinician members and diverse representation in terms of geography, age, gender, race/ethnicity, cancer diagnosis, and clinical role. Consistent with best practices in patient engagement in research [17], patient and caregiver members of the steering group are compensated for their time and expertise. 

#### 2.1.3. Research Team

An additional group of researchers with expertise in AYA cancer, patient-oriented research, and priority-setting partnerships (J.B, K.B., S.B., S.G., L.J., and M.N.) are engaged in the PSP to offer additional content and methodological guidance. Final decisions regarding PSP project activities and direction are made by the steering group.

#### 2.1.4. Project Partners

The JLA recommends that community organizations engaging in activities that reach and advocate for the target population (e.g., charities, professional organizations) should be involved in PSPs as project partners. As part of the development of the AYA Cancer PSP, the project co-leads (P.T., C.T., and F.S.) contacted all relevant patient and professional organizations in Canada and extended an invitation to join the project as a partner. Partners are asked to help disseminate information about the PSP to increase engagement in the activities of the PSP. Over 30 community organizations are engaged in the AYA Cancer PSP as partners.

#### 2.1.5. Online Presence

Key documents of the AYA Cancer PSP (e.g., JLA protocol, steering group terms of reference) are available on the JLA website [18]. A website (www.ayacancerpsp.ca (accessed on 6 May 2024)) was developed to provide further information on the project, team members, activities, and updates (e.g., quarterly newsletters). A hashtag for the project was created (#AYACancerPSP) to track posts across social media platforms.

### 2.2. Agreeing on the Scope and Protocol

The scope of a PSP is decided and agreed upon by the steering group [16]. The scope of the AYA Cancer PSP encompassed AYAs in Canada diagnosed with cancer between the ages of 15 and 39 who are living with or have recovered from cancer; caregivers of AYAs in Canada diagnosed with cancer between the ages of 15 and 39 who are living with or have recovered from cancer; bereaved caregivers of AYAs in Canada who were diagnosed with cancer between the ages of 15 and 39; and clinicians who provide direct care to AYAs in Canada diagnosed with cancer between the ages of 15 and 39.

### 2.3. Establishing the Top 10 Research Priorities

#### 2.3.1. Step 1: Gathering Uncertainties

Potential research priorities (termed “uncertainties” by the JLA [16]) are first determined through a wide-reaching online survey of patients, caregivers, and clinicians who fall within the scope of the PSP [16]. The survey for the AYA cancer PSP was launched in September 2023 and made available in both English and French. The survey was disseminated through the PSP partner organizations, the steering group’s networks, emails to pediatric and adult cancer centres across Canada, and social media (e.g., Twitter, Facebook, Instagram) and open for responses for a period of 3 months.

In the survey, AYA patients, caregivers, and clinicians were asked to describe what questions about AYA cancer they would like to see answered by research in the following areas: screening and/or diagnosis, treatment and/or care, life after cancer, end-of-life care, and other questions. As research can cover a wide range of topics including the physical, emotional, practical, and societal aspects of cancer, participants were encouraged to ask questions on any area of AYA cancer important to them, including cancer screening, clinical trials, navigating the health care system, symptoms and side effects, follow-up care, fears and concerns, family and relationships, sexual health and fertility, finances, school, work, activities, and cancer prevention. Participants were also asked to report on their demographic characteristics to allow us to describe the sample that took part.

#### 2.3.2. Step 2: Data Processing and Verifying Uncertainties

The dataset will be cleaned and organized according to JLA procedures. Each response will be given a reference number to maintain an audit trail, and data will be anonymized as needed. Responses deemed out of scope (e.g., outside the scope defined in the protocol, questions seeking advice on a topic, or issues surrounding awareness) [16] by the steering group will be removed. Eligible submissions will be categorized into groups and summarized into overarching questions. The overarching questions will be checked against existing systematic reviews and clinical practice guidelines to determine whether they have been answered or partially answered by previous research. Initial questions that have been considered answered in the last 5 years will be removed [16]. Questions that are determined to be partially answered may be revised by the steering group. Questions retained will be considered the initial “long list” of priorities.

#### 2.3.3. Step 3: Interim Priority Setting

An interim prioritization process will consolidate the “long list” of questions into a shorter list to be discussed at the final priority-setting workshop. This will be executed through an interim priority-setting online survey, disseminated using the same methods used in Step 1. Participating patients, caregivers, and clinicians will rank the 10 questions they consider to be the most critical priorities for research. Questions will be scored according to their relative rankings, and a short list of the top 20–25 questions will be established [16].

#### 2.3.4. Step 4: Final Priority Setting

A final priority-setting workshop will be held over two half-days to systematically reach a consensus on the top 10 research priorities for AYA cancer in Canada. The workshop will be held virtually to optimize accessibility and ensure participation of various individuals across Canada. Workshop participants will be 20–30 individuals who fall within the scope of the project (i.e., people with personal (e.g., patients or caregivers) and/or professional (e.g., clinicians) experience with AYA cancer) [16]. Individuals will be recruited from partner organizations, the steering committee’s networks, and contact information provided in the Step 1 and 3 surveys and through an open call. Prospective participants will be screened to ensure a balance of roles and experience as well as diversity in terms of sex and gender, race and ethnicity, age, and geographic location. Policy makers and funders may be invited to make observations [16]. Emotional support will be provided by a trained team member via telephone or private Zoom call should any participant experience distress.

The workshop will be facilitated by three trained JLA advisers to ensure fairness, accountability, and transparency. The workshop will follow an adapted Nominal Group Technique [19,20] where small and large group discussions are used to achieve consensus on the top 10 research priorities.

Prior to the workshop, participants will receive the list of the top 20–25 research questions with instructions asking them to rank these questions from highest to lowest priority [16]. During the workshop, participants will be split into small, pre-determined groups that include a balance of patients, caregivers, and clinicians. In each small group, participants will discuss their highest- and lowest-ranked research questions. Following a large group discussion, the small groups will have the opportunity to re-rank their priorities. Rankings from each small group will be combined. Participants will then be placed in new small groups to hear a wider range of opinions, where they will repeat the process of discussing the priorities and re-ordering the rankings, as appropriate. Rankings from the new small groups will be collated, and the large group will meet again to review the list and reach a consensus [16]. The workshop will result in a final list of the top 10 research priorities, establishing an actionable agenda for patient-oriented AYA cancer research in Canada.

### 2.4. Dissemination of Findings

Following the establishment of the top 10 priorities, numerous strategies will be employed to increase awareness of the priorities among researchers, patients, caregivers, clinicians, decision makers, and funders and generate new funding opportunities and proposals that address these priorities. These include presentations at national conferences and meetings; open access publication of the top 10 research priorities in a peer-reviewed journal (in accordance with the Reporting Guideline for Priority Setting of Health Research [20]); development of media and multimedia tools; sharing of the research priorities over social media using the project hashtag (#AYACancerPSP); partnering with community organizations and charities engaged in the project to establish funding opportunities to address the top 10 priorities; discussions with policymakers and health system decision makers to review existing barriers and action the identified priorities; and facilitation of ongoing collaboration between knowledge users to develop research proposals based on the top 10 priorities. Dissemination activities will acknowledge the potential limitations of the study (e.g., surveys being limited to English- and French-speaking individuals with internet access). Future work may also focus on evaluating key metrics (e.g., funding for AYA cancer research) following the establishment of the top 10 priorities [21].

## 3. Study Status

The first steering group meeting was held in April 2023. The initial survey for gathering uncertainties (Section 2.3.1) was launched in September 2023 and closed in December 2023. Data processing (Section 2.3.2) is ongoing. The JLA estimates that the PSP process takes approximately 12–18 months. [16] The final priority-setting workshop is expected to take place in fall 2024.

## 4. Conclusions

This manuscript describes the early development and study protocol for the AYA cancer PSP. Following the JLA methodology, the AYA cancer PSP will result in a top 10 list of research priorities identified by AYA patients, caregivers, and clinicians. The establishment and dissemination of a patient-oriented research agenda will catalyze a long-term and impactful AYA cancer research program in Canada. This shift to focusing on patient-, caregiver-, and clinician-identified priorities will ultimately improve outcomes for AYA patients with cancer in Canada.

## Data Availability

This protocol paper does not report any data. Data sharing is not applicable to this article.
